# Redox-directed cancer therapeutics: Taurolidine and Piperlongumine as broadly effective antineoplastic agents (Review)

**DOI:** 10.3892/ijo.2014.2566

**Published:** 2014-07-28

**Authors:** HANS MÖHLER, ROLF W. PFIRMAN, KARL FREI

**Affiliations:** 1 Institute of Pharmacology, University of Zurich and Department of Chemistry and Applied Biosciences, ETH Zurich, 8057 Zurich, Switzerland; 2 Geistlich Pharma AG, 6110 Wolhusen, University Hospital Zurich, 8091 Zurich, Switzerland; 3 Department of Neurosurgery, University Hospital Zurich, 8091 Zurich, Switzerland

**Keywords:** antineoplastic agent, reactive oxygen species, apoptosis, autophagy, necroptosis

## Abstract

Targeting the oxygen stress response pathway is considered a promising strategy to exert antineoplastic activity in a broad spectrum of tumor types. Supporting this view, we summarize the mechanism of action of Taurolidine and Piperlongumine, two antineoplastic agents with strikingly broad tumor selectivity. Taurolidine enhances the oxidative stress (ROS) selectively in tumor cells. Its cytotoxicity for various tumor cells *in vitro* and *in vivo*, which includes tumor stem cells, is based on the induction of programmed cell death, largely via apoptosis but also necroptosis and autophagy. The redox-directed mechanism of action of Taurolidine is apparent from the finding that reducing agents e.g., N-acetylcysteine or glutathione impair its cytotoxicity, while its effectiveness is enhanced by agents which inhibit the cellular anti-oxidant capacity. A similar redox-directed antineoplastic action is shown by Piperlongumine, a recently described experimental drug of plant origin. Taurolidine is particularly advantageous in surgical oncology as this taurine-derivative can be applied perioperatively or systemically with good tolerability as shown in initial clinical applications.

## 1. Introduction

A redox disequilibrium has been recognized in recent years as a specific vulnerability of various tumor cells (1–3). By exacerbating the oxidative stress, pro-oxidant drugs achieve antineoplastic activity in a multitude of cancer cells (1,4–6). Non-malignant cells, with their high anti-oxidant capacity, are largely resistant to the same degree of deviation from the redox equilibrium resulting in a high selectivity of such drugs for tumor cells (2,4–7). In the present review, Taurolidine is described as a redox-directed cancer therapeutic with a broad spectrum of antineoplastic action. In first clinical application in surgical oncology, Taurolidine was well tolerated in patients with glioblastoma and gastrointestinal cancers (8–13). In support of the ROS stress pathway being an effective target for broad antineoplastic action, the anticancer activity of Piperlongumine, a plant-derived experimental agent (14,15), is briefly outlined.

## 2. Redox-directed cancer therapeutics

Cancer arises through a multistep, mutagenic process (‘oncogene dependency’) whereby cancer cells acquire a common set of properties that enable tumor cells to proliferate and disseminate metastases (16). As an adaptive response, non-oncogenic pathways such as the oxidative stress response pathways, are also affected (1,2). Reactive oxygen species (ROS), the key mediators of cellular oxidative stress involved in cancer initiation and progression, have recently emerged as promising targets for anticancer drug development. Cancer cells have been reported to harbor elevated levels of ROS and the ability to cope with chronically elevated levels of cellular stress is compromised in a multitude of cancer cells (1–3,17). This specific vulnerability of various tumor cells was termed ‘non-oncogene dependency’ or ‘non-oncogene addiction’ (1,4,14). Importantly, this dependency may not be shared by many non-transformed cells (3–5,7). Their basal ROS levels are low and their anti-oxidant capacity is robust due to the activation of Nrf2, the master regulator of anti-oxidant responses which includes the induction of anti-oxidant enzymes such as catalase or glutathione-S-transferase (3,5,7).

In keeping with this hypothesis, small molecular weight pro-oxidant drugs, which enhance the oxidative stress in tumor cells, are considered as potential antineoplastic agents (2,3,6,15). In contrast, due to the lower basal ROS levels and elevated anti-oxidant capacity, the same pro-oxidant deviation from redox homeostasis would be tolerated by non-malignant cells leading to minimal side-effects (1–3,6,14).

In the age of molecularly targeted therapy, drugs with pleiotropic actions such as redox-directed agents, frequently find limited enthusiasm based on the expectation of off-target toxic effects. However, as exemplified in this review, at least some redox-directed agents appear to act selectively on tumor cells. In addition, recent research suggests that it is exactly the pleiotropic mode of action which seems to be uniquely tailored to overcome cancer cell drug resistance originating from a redundancy of oncogenic signaling and rapid mutation (3,6).

## 3. Broad-spectrum antineoplastic activity of Taurolidine

Taurolidine, first synthesized in the 1970s (18) as bis(1,1- dioxoperhydro-1,2,4-thiadizinyl-4)methane, was originally known for its antibacterial and anti-toxin (exo/endotoxin) activity and was tested clinically in the 1980’s in the treatment of severe surgical infections, abdominal sepsis and peritonitis (10,11,13). Its antineoplastic activity became apparent in colony forming assays, in which dissociated cells were seeded at very low density and incubated for 2 to 4 weeks. Taurolidine potently prevented cell proliferation (EC_50_, 1 to 7 μg/ml) as shown for glioma cell lines (19–21) as well as *ex vivo* human glioblastoma cells (19). In addition, at higher concentrations, Taurolidine induced acute cytotoxicity (EC_50_, 40 to 80 μg/ml), tested at 24–72 h incubation, as shown for a multitude of cultured tumor cell lines such as mesothelioma (22–24), prostate (21,25), glioblastoma (19,20,26,27), ovarian (21,28), leukemia (28), colon (21,29–36), melanoma (21,37,38), osteosarcoma (40,41), pancreatic (41), lung (21), esophageal (42) and fibrosarcoma (41,43) as partly summarized by Jacobi *et al* (44). The effectiveness of Taurolidine *in vitro* was largely confirmed *in vivo* using various tumor cell lines as xenografts such as mesothelioma (23), prostate (25), ovarian (21,45), colon (29–31,34,35) and melanoma (37,46) as well as melanoma cells in a metastatic tumor model (46).

## 4. ROS-dependent cytotoxicity of Taurolidine

As first demonstrated in glioblastoma cells (19), a ROS-dependent mechanism of Taurolidine-induced cell death became apparent in many tumor cell types. Taurolidine increased the level of ROS as shown in glioblastoma (19) and mesothelioma cells (22,24). The reducing agent N-acetylcysteine (NAC) was able to block or strongly reduce the cytotoxicity in nearly all tumor cells such as glioblastoma (20), mesothelioma cells (22,24), colon carcinoma HT29 cells (47) and Chang liver cells (47) although not in fibrosarcoma HT1080 cells (47). Addition of glutathione similarly prevented cytotoxicity as shown for mesothelioma cells (22). Conversely, a reduction of the glutathione level with DL-buthionin-(S,R)-sulfoximine (BSO) enhanced the ability of Taurolidine to induce cell death as shown for glioblastoma cells (20) as well as colon and pancreas carcinoma cells (47). These results underline the central role of ROS in triggering the Taurolidine-induced programmed cell death. Molecularly, Taurolidine may interfere with regulators of redox and ROS homeostasis such as glutathione-S transferase 1.

## 5. Taurolidine and cancer stem cells

In most tumors, the hierarchical model of tumor formation is thought to be operative with cancer stem cells (CSC) contributing to self-renewal and regrowth after debulking of tumor mass by surgery or radiation (16) e.g., in glioblastoma (48). Taurolidine exerted potent cytotoxic activity against murine and human glioma CSCs with ED_50_, 12±2 μg/ml and EC_50_, 13±2 μg/ml, respectively. The CSCs were isolated by the formation of neurospheres from either the murine SMA 560 glioma cell line or from tissue resected from newly diagnosed WHO grade IV glioblastoma patients (KF, unpublished data). These results extend the effectiveness of redox-directed cytotoxicity to CSCs and may strengthen the therapeutic potential of Taurolidine.

## 6. Sparing of normal cells by Taurolidine

Non-tumor cells *in vitro* such as bone marrow cells (28), NIH-3T3 fibroblasts (21), non-neoplastic mesothelial cells (22) were not affected by Taurolidine under conditions of tumor cell cytotoxicity. Similarly, *in vivo*, physiological cell proliferation such as leukopoiesis or erythropoiesis (28,31,44) were practically not affected by Taurolidine. The apparent selective induction of cytotoxicity in cancer cells distinguishes Taurolidine from other molecules that partly affect ROS levels, such as paclitaxel, bleomycin, cisplatin or the glutathione synthesis inhibitor BSO (6,49–53).

## 7. Good tolerability of Taurolidine in patients

Initially, Taurolidine was tested as an intraperitoneal and intravenous adjunct in the treatment of severe surgical infections (sepsis, peritonitis, pancreatitis), exploiting its activity against antibiotic-resistant bacteria and bacterial toxins. Taurolidine showed good tolerability (10,11,13). In the first clinical experiences with cancer patients in surgical oncology, Taurolidine was likewise well tolerated. In a first case report, a patient with gastric cancer re-recurrence was palliatively treated with 2% Taurolidine i.v. for 39 cycles, each cycle consisting of 7 days of treatment per month (300 mg/kg body weight per day). The patient was in good clinical condition as shown by the relevant blood parameters which included an undisturbed leukopoesis and thrombopoesis and no sign of toxicity (9). In a clinical experience with two patients with progressive, non-resectable glioblastoma and conventional therapy, the neurological condition and the quality of life improved in both patients with no sign of tumor progression (‘partial remission’) following two cycles of 21 days each with 2% Taurolidine i.v. (20 g/day) (8). In 11 patients with progresssive metastatic melanoma, co-administration of Taurolidine with high rIL-2 enhanced the tolerability of this regime (54). In a multicenter prospective randomized trial, patients with different resectable gastrointestinal (GI) cancers (20 patients each with colon, pancreas or stomach cancer) were treated with a perioperative lavage (2×10 min) consisting of 0.5% Taurolidine/heparin versus 0.25% povidone- iodine (control). Taurolidine resulted in a reduction of inflammatory cytokines (IL-1, IL-6, IL-10) at 2 and 6 h as measured in peritoneal fluid compared to pre-resection levels (12). There was no change in serum leukocytes and the perioperative complications did not differ. Up to now, the number of GI cancer patients in each group has remained insufficient for a statistical analysis of disease outcome following Taurolidine treatment (12). Nevertheless, in these oncological surgical interventions, Taurolidine did not interfere with post-operative wound healing, which was also demonstrated in a study on the use of Taurolidine in coronary artery bypass grafting in 60 patients (55). These findings confirmed previous findings in rats in which the scar tissue biopsies were examined macroscopically and histopathologically following Taurolidine treatment (56). The perioperative use of Taurolidine solution (2%) in surgical oncology (57) promises to be of special benefit as it is administered at the earliest possible therapeutic time window. Perioperatively, circulating tumor cells, which correlate negatively with disease free survival and overall survival (58), would be the prime targets for Taurolidine. By its ability to be cytotoxic to tumor cells and tumor stem cells, perioperative Taurolidine promises to reduce micrometastases and increase survival, as substantiated in a pancreatic cancer model (59). The majority of clinical studies relates to the intraperitoneal administration of Taurolidine, especially in the setting of peritonitis (11,13). In intravenous studies, vein irritation at high doses has been experienced, necessitating direct central administration or peripherally via a PICC line (57).

## 8. Potency of action and plasma levels in patients

Due to its short half-life in man (60) Taurolidine is usually administered by intravenous infusion (2% Taurolidine). When administered i.v. intermittently to glioblastoma patients, Taurolidine reached a maximal plasma level of 83±18 μg/ml (61), which is similar to peak values obtained in acutely treated healthy volunteers (60). This plasma concentration is expected to be clinically effective. It is about 20 times higher than the antiproliferative effective concentration of Taurolidine and is in the range of its cytotoxic potency.

## 9. Mechanisms of antineoplastic action

**Figure 1:**
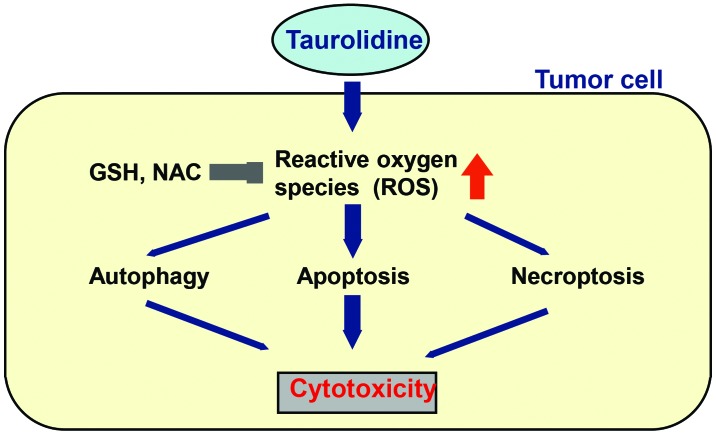
The proposed antineoplastic action of Taurolidine. By increasing ROS, Taurolidine induces cytotoxicity in tumor cells largely by induction of apoptosis, but also autophagy and necroptosis. The degree to which these processes are involved may vary with the type of tumor cell. Reducing agents such as N-acetylcysteine (NAC) or glutathione (GSH) inhibit cytotoxicity, which supports the mechanism of redox-directed antineoplastic activity.

The ROS-dependent induction of programmed cell death by Taurolidine is based on a mixed type of cellular signaling, in particular the induction of caspase-independent apoptosis but also autophagy and programmed necrosis (necroptosis) (Fig. 1).

### Apoptosis

**Figure 2:**
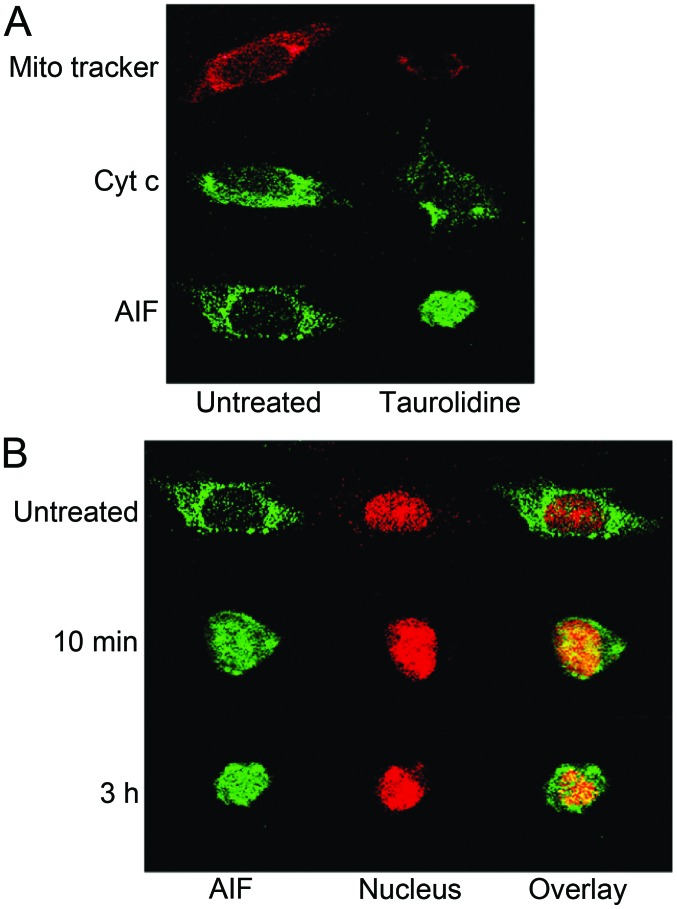
Initiation of the mitochondrial response pathway by Taurolidine (19). (A) Confocal microscopy images demonstrating Taurolidine-induced loss of mitochondrial membrane potential (red, visualized by MitoTracker) and translocation of Apoptosis-Inducing-Factor (AIF, green) from mitochondria to the nucleus in LN229 glioma cells after 2 h of Taurolidine treatment with 100 μg/ml. In contrast, cytochrome *c* (represented by the green punctate immunostaining) was not released. (B) Time dependency of the Taurolidine effect. AIF translocation, cell shrinkage, and nuclear condensation (chromatin stained red with PI) are detectable within 10 min after Taurolidine (100 μg/ml) treatment. For details see ref. 19.

**Figure 3:**
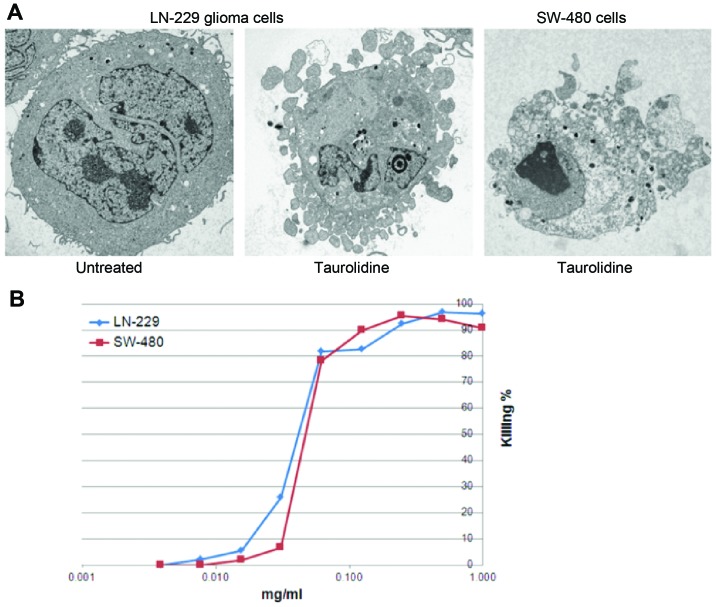
(A) Ultrastructural evidence of Taurolidine (100 μg/ml, 12 h of treatment)-induced apoptosis in LN229 glioma cells and SW-480 colon adenocarcinoma cells as shown by blebbing of the plasma membrane and chromatin condensation. (B) Dose response of Taurolidine-induced cell death as shown for the human LN229 glioma and SW-480 colon adenocarcinoma cell lines. Electron microscopy and cytotoxicity were performed as described in ref. 20.

Induction of apoptosis by Taurolidine was first shown in ovarian tumor cells (21), also in mesothelioma cells (1,24) but was most extensively studied in glioma cells (19,20,26,27). Within minutes of incubation with Taurolidine, the ROS-induced mitochondrial stress signaling pathway was activated as shown by the depolarization and permeabilisation of the mitochondrial membrane of glioblastoma cells (Fig. 2). Concomitantly, as an inducer of apoptosis, the mitochondrial apoptosis-inducing factor (AIF) was transduced from the cytoplasm into the nucleus (Fig. 2) (19). In keeping with the redox-directed mechanism, this process was completely blocked by co-incubation with N-acetyl-cysteine (NAC) (19). This reducing agent prevented the Taurolidine-induced cell death in practically all tumor cells tested, as described above, supporting the view that induction of apoptosis is the main mechanism of Taurolidine-induced cytotoxicity (19,22,24,47). Other markers of apoptosis included condensation of chromatin, fragmentation of DNA, externalization of phosphatidylserine and blebbing of the plasma membrane (Fig. 3) (19–21,23,24,27).

On the molecular level, the signaling pathways activated by Taurolidine, included the expression of pro-apoptotic transcription factors, the downregulation of the anti-apoptotoc regulator Bcl2, as well as the induction of genes involved in the ER stress response, in protein ubiquitination and in mitochondrial apoptotic pathways (42,62,63). Akt (but not Erk1,2) was inhibited (22). Taurolidine acted synergistically with TRAIL-induced apoptosis (42,63). In keeping with the ROS-dependent mechanism of action, Taurolidine was effective independent of whether p53 was mutated or not (19,20). A significant effect on DNA repair (PARP) was excluded, since PARP inhibition did not interfere with the cytotoxicity of Taurolidine (19,20).

Caspase-dependent pathways of apoptosis played a minor role. In glioblastoma cell lines, but not in *ex vivo* glioma cells, cytochrome *c* translocation was observed only to a very small extent (19,20). Nevertheless, after long-term incubation with Taurolidine (up to 48 h), some cytochrome *c*-dependent caspase activation (caspase 8 and 9) was apparent in prostate, colon and mesothelioma tumor cell lines since cytotoxicity was partly inhibited by a pan-caspase inhibitor (23,41,45). The primary molecular targets of Taurolidine remain to be identified.

### Autophagy

States of cellular stress, including ROS formation, are known to be strong inducers of autophagy, a caspase-independent process of cell death. In this lysosomal process, cytoplasm and intracellular organelles are sequestered into autophagosomes and delivered to lysosomes for degradation. In glioblastoma cells, incubation with Taurolidine (6–24 h) induced autophagy in part of the cells as visualized by the sequestration and lysosomal degradation of intracellular oganelles using transmission electron microscopy (20,41). Autophagosomes were also detected by confocal microscopy (20). Inhibition by 3-methyl-adenine is likewise in keeping with autophagy (20). Autophagy represents an alternative mechanism of cytotoxicity in particular for apoptosis-resistant tumor cells (64).

### Necrosis

The degree of Taurolidine-induced necrosis was variable. It was negligible in glioma cells but appeared more prevalent in pancreatic and fibrosarcoma cell lines (47). In glioma cell culture (LN229), Taurolidine within 24 h killed 90% of the cells as shown by the complete dissolution of the cell morphology (phase contrast microscopy before Annexin-V and PI staining). Of these cells, 53% were apoptotic, only 4.6% were necrotic (19,20). The latter was largely due to programmed necrosis (necroptosis) since pretreatment with necrostatin-1, a selective inhibitor of the receptor-interacting protein kinase RIP1, had some protective effect (about 40%) (20). The large number of unstained glioma cells (42%) may point to effects of Taurolidine beyond the induction of apoptosis and necrosis such as autophagy, as described above.

## 10. Supportive anti-angiogenic and anti-inflammatory activities of Taurolidine

Tumors are able to create a permissive microenvironment which includes the ability to induce neo-angiogenesis for maintaining the supply of oxygen and nutrients (16). Besides the induction of the programmed cell death, the antineoplastic activity of Taurolidine includes the inhibition of neo-angiogenesis. Taurolidine inhibited the adhesion of cultured endothelial cells (65), reduced the synthesis of VEGF but not of IL-6 (19,31) and potently inhibited the VEGF-induced formation of new blood vessels from human endothelial cells *in vitro* (lowest active concentration 1.25 μg/ml) (65). It remains to be seen, wether Taurolidine, besides its cytotoxic effect on tumor cells, may interfere with the neovascularization of tumors *in vivo*.

Taurolidine also showed anti-inflammatory activity as demonstrated early on by the suppression of *E. coli* endotoxin-induced endotoxemia (66) and the endotoxin-induced increase in IL-1β and TNFα synthesis in human peripheral blood monocytes (67) as well as the suppression of the stimulated release of IL-1β from peritoneal macrophages (34). There is a strong link between chronic inflammation and cancer, and NFκB is implicated as a key component in inflammation-induced tumorigenesis (68). In potentially attenuating this process, Taurolidine upregulated the NFκB inhibitor NFκBIA in fibrosarcoma and esophageal cancer cells (42,63). NFκB also regulates the release of the proinflammatory mediators IL-1, IL-6 and TNFα. The anti-inflammatory effect of Taurolidine was proposed to contribute, at least partially, to the attenuation of perioperative tumorigenesis by diminishing the surgery-related inflammation as shown in a rat melanoma model (46,57).

### 11. Piperlongumine, a broad-spectrum antineoplastic agent

**Figure 4:**
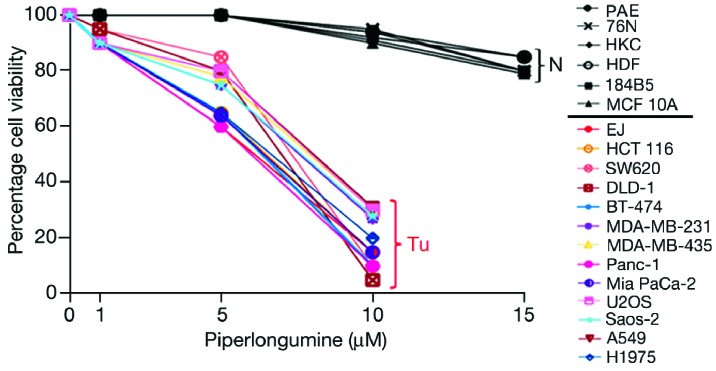
Piperlongumine treatment induces cell death in cancer cells but not in normal cells. Normal human cells (N), including aortic endothelial cells (PAE), breast epithelial cells (76N), keratinocytes (HKC) and skin fibroblasts (HDF), as well as two immortalized breast epithelial cell lines (184B5 and MCF 10A), were grown in 12-well or 24-well plates and treated with piperlongumine at 1–15 μM for 24 h. A variety of human cancer cell lines (Tu) were also treated with piperlongumine or DMSO (control) for 24 h. Cytotoxicity was measured by trypan blue exclusion staining (average of three independent experiments) (reproduced with permission from ref. 14).

The plant alkaloid Piperlongumine (PL), which was previously reported to have antibacterial properties (69), was recently shown to display broad antineoplastic activity by targeting the ROS stress pathway in tumor cells. PL caused a marked increase in ROS selectively in cancer cells as shown in four cancer cell lines (incubation for 1 and 3 h) (14). PL dose-dependently induced cytotoxicity in all 13 different tumor cell lines tested with half maximal effects being reached at 6–8 μM (Fig. 4) (14). The increase in ROS in tumor cells and the cytotoxicity of PL was reduced by co-incubation with the enzyme catalase or blocked by the addition of NAC (14). PL is thought to interfere with redox and ROS homeostatic regulators such as glutathione-S transferase 1 or carbonyl reductase (14). PL, under comparable conditions, did not cause an increase of ROS or cytotoxicity in normal cells such as endothelial cells, breast epithelial cells, keratinocytes and skin fibroblasts (Fig. 4) (14). The PL-induced killing of a broad spectrum of tumor cell lines was based on a mixed type of cellular signaling based largely on the induction of apoptosis (70) but also of autophagy. The latter was triggered via the p38 protein kinase ROS stress response pathway (71). In prostate cells, inhibition of proliferation included the downregulation of the transcription factor NFκB (72). Thus, PL is an interesting broad-spectrum, redox-directed experimental antineoplastic agent.

### 12. Conclusions

Taurolidine is a representative of a novel class of redox-directed, broad-spectrum antineoplastic agents with tumor-selective cytotoxicity. It induces programmed cell death by targeting the oxidative stress response pathway which is compromised in many tumor cells. In initial applications in surgical oncology, Taurolidine i.v. was largely free of significant side-effects and merits further clinical evaluation. Applied perioperatively, Taurolidine promises to reduce circulating tumor cells, a negative predictor for disease-free survival. Taurolidine also inhibits VEGF-induced neo-angiogenesis and may therefore display a dual mode of antineoplastic action. Piperlongumine, an experimental plant-derived agent, is another redox-directed broad-spectrum antineoplastic agent with tumor cell selectivity.
